# The Role of Relationship Dynamics and Gender Inequalities As Barriers to HIV-Serostatus Disclosure: Qualitative Study among Women and Men Living with HIV in Durban, South Africa

**DOI:** 10.3389/fpubh.2017.00188

**Published:** 2017-07-31

**Authors:** Divya S. Bhatia, Abigail D. Harrison, Muriel Kubeka, Cecilia Milford, Angela Kaida, Francis Bajunirwe, Ira B. Wilson, Christina Psaros, Steven A. Safren, David R. Bangsberg, Jennifer A. Smit, Lynn T. Matthews

**Affiliations:** ^1^Department of Behavioral and Social Sciences, Brown University School of Public Health, Providence, RI, United States; ^2^Maternal Adolescent and Child Health Research Unit, Faculty of Health Sciences, Department of Obstetrics and Gynaecology, University of the Witwatersrand, Durban, South Africa; ^3^Faculty of Health Sciences, Simon Fraser University, Burnaby, BC, Canada; ^4^Mbarara University of Science and Technology, Mbarara, Uganda; ^5^Department of Health Services, Policy and Practice, Brown University School of Public Health, Providence, RI, United States; ^6^Behavioral Medicine Program, Massachusetts General Hospital, Department of Psychiatry, Boston, MA, United States; ^7^Harvard Medical School, Boston, MA, United States; ^8^Department of Psychology, University of Miami, Miami, FL, United States; ^9^Oregon Health Sciences University, Portland, OR, United States; ^10^Portland State University School of Public Health, Portland, OR, United States; ^11^Discipline of Pharmaceutical Sciences, College of Health Sciences, University of KwaZulu-Natal, Durban, South Africa; ^12^Massachusetts General Hospital, Division of Global Health, Boston, MA, United States; ^13^Massachusetts General Hospital, Division of Infectious Diseases, Boston, MA, United States

**Keywords:** gender inequality, partner communication, qualitative, HIV-serostatus disclosure, barriers to disclosure, couples-based HIV counseling and testing, relationships, living with HIV/AIDS

## Abstract

**Background:**

This qualitative study investigated gender power inequalities as they contribute to relationship dynamics and HIV-serostatus disclosure among men and women living with HIV in Durban, South Africa. HIV serodiscordance among men and women within stable partnerships contributes to high HIV incidence in southern Africa, yet disclosure rates remain low. Given the emphasis on prevention for HIV-serodiscordant couples, this research supports the urgent need to explore how best to support couples to recognize that they are part of this priority population and to access appropriate prevention and treatment.

**Methods:**

Thirty-five in-depth individual interviews were conducted with 15 HIV-positive men and 20 HIV-positive women (not couples) receiving care at public-sector clinics near Durban. A structured coding scheme was developed to investigate men’s and women’s attitudes toward HIV-serostatus disclosure and behaviors of sharing (or not sharing) HIV serostatus with a partner. Narratives were analyzed for barriers and facilitators of disclosure through the lens of sociocultural gender inequality, focusing on reasons for non-disclosure.

**Results:**

Among 35 participants: median age was 33 years (men) and 30 years (women); average years since HIV diagnosis was 1 (men) and 1.5 (women). Four themes related to gender inequality and HIV-serostatus disclosure emerged: (1) Men and women fear disclosing to partners due to concerns about stigma and relationship dissolution, (2) suspicions and mistrust between partners underlies decisions for non-disclosure, (3) unequal, gendered power in relationships causes differential likelihood and safety of disclosure among men and women, and (4) incomplete or implicit disclosure are strategies to navigate disclosure challenges. Findings illustrate HIV-serostatus disclosure as a complex process evolving over time, rather than a one-time event.

**Conclusion:**

Partner communication about HIV serostatus is infrequent and complicated, with gender inequalities contributing to fear, mistrust, and partial or implicit disclosure. Relationship dynamics and gender roles shape the environment within which men and women can engage successfully in the HIV-serostatus disclosure process. Integrated interventions to reduce barriers to trustful and effective communication are needed for HIV-affected men and women in partnerships in which seeking couples-based HIV counseling and testing (CHCT) is challenging or unlikely. These data offer insights to support HIV-serostatus disclosure strategies within relationships over time.

## Introduction

In South Africa, young women are disproportionately at risk for HIV (1–4); HIV prevalence increases from 7% among women aged 15–19 to 17% at ages 20–24, compared to 0.7 and 5% among men in those age groups, respectively (1, 2, 5). Rates of HIV serodiscordance within couples—wherein one partner is HIV positive and the other partner is not—are estimated at 25% in South Africa (6, 7), contributing to sustained high HIV incidence (3, 4). Despite research informing and promoting public health strategies to support prevention for HIV-serodiscordant couples in this setting, rates of HIV-serostatus disclosure remain low among both men and women, hindering access to prevention (8–11).

HIV-serostatus disclosure encompasses the process and experience of sharing one’s HIV infection status with others (12, 13). This process can facilitate couples’ access to available HIV treatment and prevention options (14). Studies suggest that men and women who communicate with their partner about HIV-serostatus are more likely to seek out and adhere to antiretroviral therapy (ART) (15–17), cope with their diagnoses (18), seek increased social support (19), and engage in protective behaviors including condom use (20, 21). However, difficulties surrounding disclosure communication may prevent the use of HIV prevention methods or result in suboptimal adherence to HIV treatment (14, 22, 23). HIV-serostatus disclosure may be particularly stressful for women due to fear of negative reactions from one’s partner upon disclosure (24–26), including violence (27–29), discrimination, abandonment, or accusations of infidelity (24, 26, 29, 30). Safer disclosure strategies are needed (31), including harm reduction approaches (32), especially during pregnancy when women are often more vulnerable (27, 33–36). In addition, given the emphasis on prevention for HIV-serodiscordant couples (8–11), data are needed to explore how to best support couples to recognize they are part of this priority population and access prevention and treatment services.

Socially and culturally rooted gender power inequality within relationships and intimate partner violence place South African women at increased risk of HIV infection compared to men (3, 4, 33). South African gender norms are rooted in sociocultural expectations and historical contexts of violence and oppression (33), resulting in men often leveraging more power in sexual partnerships (3, 9, 33). Gendered social norms that enable male power in sexual relationships also include intergenerational relationships between younger women and older men (4, 9). Women may experience difficulty negotiating safer sex practices (33), or communicating about intimacy (37), adding to the difficulty of discussing HIV serostatus or similar topics (20, 21, 37–39). The intersection of HIV and gender power inequality within relationships has been explored and analyzed extensively as an important sociocultural determinant of HIV risk (33, 34), yet the implications for HIV-serostatus disclosure have not been comprehensively explored (24). Research from Uganda and Zimbabwe explored the process and implications of HIV-serostatus disclosure between sexual partners (12, 18, 20, 28, 39). However, the nuanced barriers and strategies to disclose have not been adequately investigated in this population of South African men and women living with HIV, and their partners (10, 14, 17), creating the need for a qualitative investigation.

We used qualitative methods to explore dynamics of HIV-serostatus disclosure, and associated barriers and promoters, to inform strategies for safe disclosure among HIV-infected South African men and women. We investigated the process, experiences, and consequences of HIV-serostatus disclosure through the lens of gender inequality by exploring HIV-infected men’s and women’s narratives of non-disclosure within relationships. By exploring how gender roles and relationship dynamics influence the disclosure process, we offer insights to inform future interventions.

## Materials and Methods

### Setting and Participant Recruitment

Data were collected within a study exploring reproductive decision-making and safer conception counseling experiences to safely address fertility goals among men and women living with HIV in eThekwini district, KwaZulu-Natal (40, 41). In this region, HIV prevalence among pregnant women attending antenatal services is estimated at 41% (42).

Individual in-depth interviews were conducted in June and July 2012 with HIV-infected men (*n* = 15) and women (*n* = 20) (not couples) enrolled in HIV care in one of four public-sector health clinics. Eligible participants were aged 18–40 years (women) or over 18 years (men), self-reported being HIV positive, were not pregnant (women), and spoke English or isiZulu.

### Ethics and Regulatory Approvals

Ethics approvals were obtained from University of the Witwatersrand Human Research Ethics Committee (Johannesburg, South Africa) and Partners Healthcare (Boston, MA, USA). Permissions were obtained from local provincial and district Departments of Health and the individual health facilities. All participants provided written informed consent.

### Data Collection

Open-ended in-depth interviews lasted approximately 1 h and explored participant experiences of reproductive goals, lived experiences of HIV, HIV-serostatus disclosure, and relationships. Interviews were conducted by research assistants fluent in English and isiZulu. Interviews were digitally recorded and transcripts were translated into English. Transcripts were reviewed for translation quality and fidelity by another study team member.

### Data Analysis

The findings were compared and contrasted across participants and genders using a rigorous analytical process to establish robust qualitative results. Themes relating to promoters and barriers of HIV-serostatus disclosure were identified and explored, based on a conceptual framework developed to guide analytical decisions considering how gender inequality shapes partnership dynamics that influence HIV-serostatus disclosure behaviors. Transcripts were read to identify major themes, analyze parallels across men’s and women’s experiences, and inform the development of a coding scheme to categorize data. Multiple coders engaged in an iterative analytical process to ensure that codes were developed using a structured, consensus-driven process. The final coding scheme included both *a priori* themes and those developed from preliminary readings of the transcripts (43). Data were organized using NVivo 10 (QSR International) and separated into themes and subthemes relating to barriers and promoters of HIV disclosure. Data reduction methods were employed to extract the overarching narrative from the most pertinent data (44).

### Conceptual Framework

**Figure 1:**
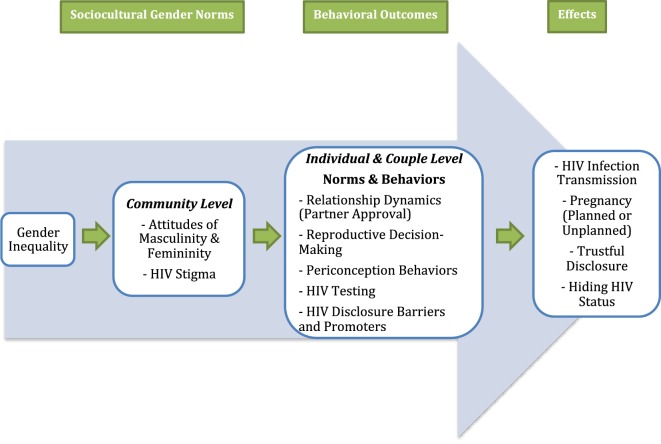
Conceptual framework diagram.

The critical analysis framework (Figure 1) contextualizes HIV-serostatus disclosure within community-level gender norms in South Africa. It examines the intersections between South Africa’s HIV/AIDS epidemic and the realities of gender inequality. This framework identifies sociocultural gender inequality as the root cause of the higher rates of HIV infection among women through its influence on individual and couple-level behaviors and partnership dynamics. In turn, these gendered behavioral outcomes influence HIV-serostatus disclosure as well as decisions surrounding conception and childbearing that place women at higher risk of HIV infection than men (4). The coding scheme contextualized gender-specific data within this framework to analyze how gender inequalities influence the process of HIV-serostatus disclosure within relationships.

## Results

The study population (*n* = 35) had the following characteristics: median age 33 years (men) and 30 years (women); average years since HIV diagnosis 1 (men) and 1.5 (women); 60% of men and 65% of women were on ART. Although a large proportion (11/15 men and 16/20 women) reported having disclosed their HIV serostatus, almost half of women did not know their partner’s HIV serostatus (Table 1).

**Table 1:** Sociodemographic characteristics of participants.

	Men (*n* = 15)	Women (*n* = 20)
Median age (IQR)	33 years (28.5–38.5)	30 years (27–33.3)
**Employment**		
Employed	8 (53%)	8 (40%)
Unemployed	7 (47%)	11 (55%)
Student	0	1 (5%)
Average years since HIV diagnosis (IQR)	1 (0.4–2.2)	1.5 (0.9–6.0)
**Currently on antiretroviral therapy**		
Yes	9 (60%)	13 (65%)
No	5 (33%)	7 (35%)
**Disclosed to current sexual partner**		
Yes	11 (73%)	16 (80%)
No	2 (13%)	3 (15%)
N/A (no current relationship)	2 (13%)	1 (5%)
**HIV status of primary partner**		
HIV positive (seroconcordant)	9 (60%)	8 (40%)
HIV negative (serodiscordant)	2 (13%)	2 (10%)
“Do not know”	2 (13%)	9 (45%)
N/A (no current relationship)	2 (13%)	1 (5%)

### Overview

Four major themes regarding HIV-serostatus disclosure emerged. First, men and women fear HIV-serostatus disclosure to partners due to concerns about stigma and potential relationship dissolution. Second, suspicions and mistrust between partners underlie and contribute to lack of disclosure. Third, unequal power in relationships based on gender influences women’s disclosure patterns, resulting in different disclosure practices for men and women. Fourth, these factors often lead to partial or incomplete disclosure. These findings reveal how men, women, and their partners experience HIV-serostatus disclosure as a complex process rather than a one-time event, and highlight important considerations for interventions.

#### Men and Women Fear Disclosing to Partners

HIV-serostatus disclosure was recognized as an important “first step” (30- to 34-year-old female) to caring for oneself and one’s partner, although both men and women experienced tension with the process.

If a person is scared to say they are living with HIV…maybe the person she met is HIV positive…or both of them think they are negative. When one of them is positive, one might end up getting infected because they did not tell each other the truth. (30- to 34-year-old female)

Participants feared consequences of disclosure, including stigmatization, accusations of infidelity, loss of a partner, and violence. As one man described:

Being sick like this, I will date someone and disclose to her and she will just leave. (40- to 44-year-old male).

One woman’s “*husband left [her] with their children*” after learning she was HIV infected (35- to 39-year-old female), while another expressed fear of “*what kind of person he [her partner] would be*” upon learning her serostatus (30- to 34-year-old female). Above all, participants feared being unable to live a normal life, inclusive of intimate relationships. Comments reflecting community stigma, such as “*most people are scared to be HIV positive*” (25- to 29-year-old female), or people think HIV “*mean[s] that, [it] is the end of your life*” (30- to 34-year-old male) were common.

Most frequently, participants who disclosed did so because they did not “*want [their partner] to get infected*” (30- to 34-year-old male), although motivations sometimes differed by gender. Pregnant women often disclosed to secure partner support to seek health care to prevent perinatal transmission and relationship dissolution.

If you didn’t [disclose], you are killing your child because the child will be infected while you were scared to come forward. (35- to 39-year-old female)

In contrast, some men feared that disclosure might interfere with their reproductive goals.

To have more children…I would impregnate someone who doesn’t know I’m [HIV-infected]. (40- to 44-year-old male)

These responses characterize gender differences in the approach to HIV-serostatus disclosure.

#### Suspicions and Mistrust within Relationships Underlie Lack of Disclosure

Both men and women viewed trust as the foundation for HIV disclosure within a relationship, yet frequently described partners as not “trustworthy” (40- to 44-year-old male).

Men have a problem…They are scared…to come forward even if they know their status. They are ruining lives because they want to infect you without [disclosing to] you. (35- to 39-year-old female)

Some participants feared that their partner had not been truthful when disclosing:

I cannot say she was being truthful when she said she’s negative. Women, especially, say they are negative, even if [they] are positive. (25- to 29-year-old male)

This man’s concerns about his partner influenced his own decision not to disclose.

Male respondents’ suspicions of women as untrustworthy were often linked to infidelity:

I can…tell my partner that I have the virus …[but] at the end if she got sick she wouldn’t know if she got it from me or from another person because women, they are not trustworthy sometimes. Also us men, we do not trust ourselves. (40- to 44-year-old male)

I have some doubts if it is me who made [my girlfriend] pregnant. (40- to 44-year-old male)

With women perceived as devious or subversive, conversations about condom use or HIV prevention were viewed as trickery, rather than honest attempts to disclose. Some men implied that women’s non-use of condoms or other HIV prevention was willful, aimed at intentionally spreading HIV infection, such that they “deserved” to be infected (30- to 34-year-old male). At the same time, men and women both expressed concerns about infecting their partner, and also about the importance of HIV disclosure to prevent this.

Further, both men and women feared that condom use would mark them as HIV infected. Secretive behaviors were common: “*He does not know I am on family planning*” (25- to 29-year-old female). One woman’s partner stated that her initiation of HIV treatment while he did not would “destroy him somehow” (35- to 39-year-old female), conveying a sense of mistrust that also precluded ARV use. Accordingly, one participant observed, “*[Many women] who are taking ARVs hide that they are taking [them]*” (35- to 40-year-old female). Others described how they exist in limbo concerning their own and their partner’s HIV serostatus: “*I don’t know [my partner’s status], that’s the main thing we’re fighting over*…*me too, I felt I must not tell him [my status]*” (30- to 34-year-old female).

In contrast to these prevailing attitudes, some women and men described communication about disclosure as a way to “respect each other” (30- to 34-year-old female) and maintain a faithful, honest relationship. As two participants described:

As people who are positive, you must be faithful to your partner that you love. You have to be open about your status. You tell him and he tells you. (30- to 34-year-old female)

She knows mine [HIV-serostatus] and I am in this situation now because she…encouraged me to get tested. (30- to 34-year-old male)

Men and women who described disclosure as beneficial often viewed it as a means of communication, whereby “*no one gets discriminated between the partners*” (35- to 39-year-old male).

#### Unequal Power in Relationships Influences Gendered Disclosure Practices

Although women appeared more accepting of disclosure, they were generally more affected by gender inequality within relationships and more concerned about negative consequences. Because of mistrust, stigma, and the potential loss of a relationship and its social and economic security, many women lived with partners for some time without disclosing: “*I am scared to tell him [my partner] I am HIV positive*” (25- to 29-year-old female).

Often, this silence was based on fears of how a partner might react, including accusations of “bringing HIV into the relationship,” reflecting respondents’ concerns about infecting their partners as well as the negative reactions that might result:

I knew [for] ten years that I was positive. I was unable to tell him. I asked him to go and check and he came back with results showing he was negative. I was unable to tell him I am positive because I was thinking what he was going to say, from where I got this. (35- to 39-year-old female)

Another woman described a 12-year relationship, in which she did not know her partner’s status while he knew she was living with HIV (30- to 34-year-old female), and she felt that asking him might disrupt the relationship.

Participant: This is the twelfth year [we are living together]…I don’t know his status.

I: Does your partner know your HIV status?

Participant: Yes he knows. (30- to 34-year-old female)

Often, women were first to test for HIV, which added further stress by making them responsible for encouraging their partner to test:

I found out [about my infection] from my wife, she was the one who came first here. She tested…and found out she was positive. I came after and found out I’m positive. I’m about to start [ARVs]. (25- to 29-year-old male)

Although both men and women described HIV disclosure as stressful, women were generally viewed as more open to it. Indeed, some women appeared more comfortable with the process, reporting that “*[men] don’t want to talk about things concerning HIV*” (25- to 29-year-old female). Many women described a partner’s unwillingness to test:

I: Why did you not tell [your partner your status]?

Participant: If he agreed to come to clinic, we would find out together, I would tell him that I am already like this, go and check. (30- to 34-year-old female)

Whether they chose to disclose or not, both men and women expressed deep-seated concerns about a partner’s reaction to learning their HIV status and the implications for their relationship.

#### Incomplete Disclosure As a Strategy to Navigate Disclosure Challenges

In some cases, participants thought their partner was HIV positive and encouraged them to disclose, but reported that they “kept on denying” (35- to 39-year-old female).

[My partner] was trying to tell me indirectly about his situation [HIV-positive], but he was scared. (20- to 24-year-old female)

Participants frequently learned of their partner’s HIV-positive serostatus through unspoken clues, including physical signs and symptoms:

In order for me to become HIV positive, the condom burst. I went to check alone and came back with results to show him…There were some warts I saw on his private parts. I [knew] something is a problem. (30- to 34-year-old female)

Many participants believed that if their partner was HIV positive then they, too, must be positive. One woman explained how she sought assistance to explain serodiscordance to her partner:

I explained to her [the nurse] that I have this problem, I request you to [explain] so this male person could understand [serodiscordance], how [this infection] has been found in me, because it can happen that, it is a female person who is found positive and not the male. And sometimes, it happens that it is found in a male and not in a female. (35- to 39-year-old female)

Many men assumed that their partner was HIV negative without having been told directly.

Participant: I know her status. It is right.

*I*: …*it is negative?*

Participant: Even though she never told me, I know that she has nothing. (30- to 34-year-old male)

Sometimes, disclosure was implicit rather than explicit. In the following exchange, a woman tells her partner only that she is “sick,” without stating directly that she is HIV infected.

I: Does your partner know your HIV status?

Participant: No. But I told him that I am sick.

*I*: …*So he knows?*

Participant: He knows, yes. (35- to 39-year-old female)

Avoiding conversations about HIV status or providing untruthful responses were consequences of the fear surrounding disclosure and the fear of losing one’s partner.

When I ask him what were the results of your blood test he will say ‘hay you know,’ then I’d ask ‘what do you mean…HIV or negative?’ then he will say negative. (25- to 29-year-old female)

These vague discussions about HIV status frequently led to partial or incomplete disclosure, in which individuals were uncertain about their partner’s serostatus.

## Discussion

HIV-serostatus disclosure, a critical component of HIV prevention, is a complicated and often indirect process. This research found that (1) Partner communication about HIV serostatus is infrequent, and the gendered nature of mistrust, fear, and suspicion within relationships creates multilayered barriers to disclosure, often leading to partial or implicit disclosure; (2) participants were often uncertain about partner serostatus, reflected in vague discussions about disclosure; (3) relationships and gender roles impact HIV-serostatus disclosure by influencing the environment within which discussions about HIV-serostatus disclosure occur; and (4) multistep interventions that occur over time to facilitate the disclosure process and reduce barriers to effective communication and trust are needed for HIV-affected men and women in relationships. For many men and women in this setting, seeking couples-based HIV counseling and testing (CHCT) together would be challenging or improbable.

Couples-based HIV counseling and testing, an evidence-based strategy to promote HIV-serostatus disclosure within partnerships, has been implemented with some success in South Africa, Rwanda, and Zambia (14, 21, 45–47). While CHCT is a beneficial strategy for couples who are able to undertake HIV testing together, it is not effective for many couples given the relationship distrust and fears of disclosure highlighted in this study. CHCT can be especially challenging because it requires that both partners go together for HIV counseling and testing, and thus functions under the assumption that both partners are comfortable discussing their HIV status with each other and that they have already disclosed to each other. This expectation is not feasible for many men and women living with HIV infection. Our findings suggest a need for approaches with attention to gender and relationship dynamics, with particular attention to the fear, mistrust, and misunderstandings of serodiscordance surrounding disclosure (11–13, 18, 20, 25, 36).

Gender inequalities influence disclosure by fostering general distrust between men and women and deep fears of repercussions of disclosure. In this study, both men and women worried about infidelity, and women feared male partner violence as well as accusations about transmitting HIV, although implications of HIV disclosure differed by gender. Men feared losing their relationship and a partner with whom to have a child, while women’s concerns focused on losing the relationship itself, including social and economic support. Many women feared violence as an outcome of HIV-serostatus disclosure; developing interventions to address these fears is critical. For both men and women, the level of relationship trust necessary for disclosure was often absent. Instead, suspicions, fears, and mistrust were barriers to disclosure. Importantly, men, women, and their partners experience disclosure as a complex process that evolves over time, not a one-time event. This process is complex because conversations about disclosure consider aspects of relationships beyond partners’ serostatus alone. Couples-based strategies could be enhanced to include HIV counseling and testing as well as gender-based violence prevention and other intervention components to address gender inequalities and stigma, the issues identified as being of paramount importance in this study.

These findings highlight the gendered nature of mistrust and suspicion within relationships (11, 24). Consistent with research findings from other African settings, many women in this study felt obligated to disclose, yet simultaneously feared consequences of losing their partner (19, 20, 27, 29, 36, 38, 48) or accusations of infidelity and infecting the partner (27, 29). This led women to hide their HIV serostatus or even ART use (15, 16). Both men and women experienced HIV-serostatus disclosure as uncomfortable and stressful. These findings show that facilitators of the HIV-serostatus disclosure process include trustful and honest partner communication while barriers include stigma, gender inequalities, and mistrust within the relationship. In this study, more women had disclosed and appeared more comfortable with the process overall, especially when motivated to help their partner test and receive ART (18, 26). Correspondingly, men more often assumed that they knew their partner’s HIV status by forming conclusions based on prior instances or interactions with their partner, even without formal disclosure. Respondents had concerns about how their partners would react to their HIV disclosure, as well as broader concerns about infecting their partners.

Other research has found that gender power inequalities powerfully shape attitudes within relationships, influencing patterns of HIV disclosure. A widespread lack of communication grounded in fear and mistrust is also common in HIV-affected partnerships (9, 12, 45), as is confusion about serodiscordance. In this study, many men and women believed that if their partner was HIV infected then they, too, must be infected, a situation known as “testing by proxy” that reflects common misconceptions about HIV serodiscordance (49–52). Partly due to such misunderstandings about HIV serodiscordance, partial disclosure is common (12, 22, 31).

Our study shows how gender inequality serves as a barrier to HIV-serostatus disclosure. We found that likely facilitators of the disclosure process would be interventions that provide support for couples to address stigma, violence, and concerns about confidentiality within their relationship. Rather than approaching disclosure as a discrete, one-time event (21, 53), disclosure interventions may be more effective if they engage participants over time, especially if they are not yet prepared to seek CHCT together. Multisession interventions may be required to reduce stigma and support disclosure communication within relationships to have constructive conversations about HIV-serostatus disclosure and treatment. Strategies with known efficacy to increase communication between partners include community-based support groups for men and women (19, 37, 46, 47), which draw on psychosocial or peer-adherence models (47, 49, 54–56). Systematic reviews of HIV-serostatus disclosure interventions show that cognitive-behavioral group sessions, peer support groups, and voluntary partner notification may be effective in encouraging disclosure to sexual partners and can also impact morbidity and retention in care (31, 53). Combining such promising approaches to develop gender-focused interventions to teach disclosure strategies individually for HIV-affected men and women in partnerships who are unable to seek CHCT together would be a significant step. Multistep interventions conducted over time with individuals or single-sex groups of men and women in serodiscordant partnerships could focus on improving communication challenges identified as barriers to disclosure in this study. Potentially effective strategies to enable individuals to engage with disclosure prior to attending CHCT include facilitating role-playing scenarios, or home visits by community health workers. Behavioral approaches that incorporate gender-focused components, including gender equality and violence reduction, could be combined with interventions that address HIV stigma and barriers to honest communication to develop an integrated strategy that addresses gender inequality’s role in HIV-serostatus disclosure (14).

This study’s participants were not couples but individual heterosexual men and women discussing their relationships. There are advantages to this, however, as much can be learned about couples, with potentially greater honesty from men and women who knew their partner was not in the study. Differences in HIV disclosure among men and women, including some women’s greater comfort with the process, may reflect social desirability bias and gender differences in reporting personal experiences. This may also result from women’s greater participation in health care, usually through antenatal care, and greater likelihood of receiving HIV testing and treatment.

## Conclusion

This paper investigates how relationship dynamics and gender inequalities serve as barriers to HIV-serostatus disclosure, and attitudes and behaviors that may promote it. Relationship and gender roles shape the environment within which men and women can engage productively in the HIV-serostatus disclosure process. These findings highlight the consequences of implicit or incomplete disclosure and the fact that, despite participants’ concerns about disclosure, non-disclosure is equally serious. Multisession interventions focused on engaging individuals or couples and health-care providers over time may reduce barriers to effective and trustful communication for the many HIV-affected men and women in partnerships in which seeking CHCT together is challenging or unlikely. Combination interventions to strengthen women’s agency, and programs to change men’s attitudes toward HIV-serostatus disclosure, are interventions worthy of further testing.

## Ethics Statement

Ethics and regulatory approvals. This study was carried out in accordance with the ethics recommendations of the University of the Witwatersrand Human Research Ethics Committee (Johannesburg, South Africa) and Partners Healthcare (Boston, MA, USA). All participants provided written informed consent in accordance with the Declaration of Helsinki. The protocol was approved by the local provincial and district Departments of Health and the individual health facilities.

## Author Contributions

LM was involved in all phases of the work. AH, AK, FB, IW, CP, SS, DRB, and JS were involved in study design, tool development, interpretation of data, and editing and final approval of the manuscript. DSB led data analysis and interpretation and production of the manuscript. MK and CM were involved in data collection, interpretation, and editing and final approval of the manuscript. All the authors read and approved the final manuscript.

## Conflict of Interest Statement

The authors declare that the research was conducted in the absence of any commercial or financial relationships that could be construed as a potential conflict of interest.

## Abbreviations

ART, antiretroviral therapy; ANC, antenatal care; CHCT, couples-based HIV counseling and testing.
